# PAIN BEHAVIORS AND PAIN TREATMENTS AMONG ENGLISH- AND SPANISH-SPEAKING HISPANICS IN US NURSING HOMES

**DOI:** 10.1093/geroni/igae098.2402

**Published:** 2024-12-31

**Authors:** Natalia Nielsen, Maira Castañeda-Avila, Kate Lapane

**Affiliations:** University of Massachusetts Chan Medical School, Worcester, Massachusetts, United States; University of Massachusetts Chan Medical School, Worcester, Massachusetts, United States; University of Massachusetts Chan Medical School, Worcester, Massachusetts, United States

## Abstract

In U.S. nursing homes, racial/ethnic differences in nonverbal pain indicators and pain management have been documented among individuals with communication challenges. The role of language has yet to be explored. We evaluate the extent to which English- and Spanish-speaking Hispanics (H-E, H-S, respectively) were less likely to have nonverbal pain behaviors recorded and more likely to have no pain management relative to non-Hispanic English-speaking White residents (NHW-E). We identified newly admitted residents using national Minimum Data Set 3.0 data between 2010 and 2020 (n=1,201,724). Adjusted prevalence ratios (aPRs) and 95% confidence intervals (CI) estimated using robust Poisson models compared pain behaviors and treatments across ethnic/language groups. Vocal complaints and facial expressions were most commonly documented (Vocal: 29.1% NHW-E, 17.1% H-E, 18.5% H-S; Facial: 20.3% NHW-E, 15.0% H-E, 18.3% H-S). Documentation of pain behaviors was less frequent among Hispanic residents than NHW-E (e.g., any pain behaviors: aPR H-E: 1.17; 95% CI: 1.14-1.19; aPR H-S: 1.11; 95% CI: 1.10-1.13). Hispanic residents (H-E: 47.8%; H-S: 49.1%) were less likely to receive any type of pharmacologic pain intervention compared with NHW-E (59.4%) (aPR H-E: 0.85; 95% CI: 0.83-0.88; aPR H-S: 0.88; 95% CI: 0.86-0.90). Staff participating in the MDS assessment were less likely to document pain behaviors and its treatment among Hispanic residents relative to non-Hispanic white residents, with differences not observed based on resident’s language. Interventions to improve recognition of pain behaviors among Hispanics are warranted.

